# Evaluation of Frying Performance, Storage Stability, and In Vitro Digestion of Extra Virgin Olive Oil‐Candelilla Wax Oleogel

**DOI:** 10.1111/1750-3841.70405

**Published:** 2025-07-10

**Authors:** Elif Kutahneci, Hasan Yalcin

**Affiliations:** ^1^ Department of Gastronomy and Culinary Arts Cappadocia University Nevşehir Türkiye; ^2^ Graduate School of Natural and Applied Sciences, Food Engineering Erciyes University Kayseri Türkiye; ^3^ Department of Food Engineering Erciyes University Kayseri Türkiye

**Keywords:** CDW, deep‐fat frying, EVOO, in vitro digestion, oleogel, oxidative stability, storage stability

## Abstract

The present study investigates the stability of an oleogel developed from extra virgin olive oil (EVOO) and candelilla wax (CDW) as a frying medium, including its 90‐day storage stability under dark and light conditions and its effects on in vitro digestion. The findings of the chemical analyses indicated that the gel structure and its storage in darkness effectively reduced oxidation, whereas exposure to light accelerated oxidative degradation. In addition, the oleogel, when utilized as a frying medium, exhibited superior stability compared to EVOO. Furthermore, the oil absorption rate of sliced potatoes fried in EVOO was 14.51%, which was lower (*p* < 0.05) at 10.29% in potatoes fried in the oleogel. It was observed that during in vitro digestion, the gel structure reduced the interaction between lipase and oil, thereby slowing the formation rate of free fatty acids (FFA). As a result, the lipolysis rates in EVOO and the oleogel were 54% and 44%, respectively. Following in vitro digestion, α‐tocopherol bio accessibility was determined to be 98% and 94% in EVOO and the oleogel, respectively, while bioavailability rates were 16% and 34%, respectively. The bio accessibility of β‐carotene was found to be 53% and 41% for EVOO and the oleogel, respectively, while the bioavailability of β‐carotene was 6% and 3%, respectively. These results indicate that the oleogel structured with CDW offers significant advantages in terms of storage stability and effectiveness as a frying medium. At the same time, the gel structure demonstrated a notable influence on the digestion of the components in EVOO.

**Abbreviations**
CDW: candelilla wax
DO: Dark‐stored oleogel
DOO: Dark‐stored extra virgin olive oil
EVOO: extra virgin olive oil
FFA: free fatty acids
FTIR: Fourier Transform Infrared
LO: Light‐stored oleogel
LOO: Light‐stored extra virgin olive oil
OBC: oil binding capacity
p‐AV: p‐anisidine
PV: peroxide value
XRD: X‐ray diffractometry

## 1 Introduction

A food's nutritional value and functional properties vary significantly depending on the type of fat used in the food. In addition to providing energy, fats and oils support body functions by facilitating the absorption of specific vitamins. Solid fats used in processed foods are one of the most critical components used in foods, as they provide the desired sensory and rheological properties while exhibiting better oxidative stability and longer shelf life (Manzoor et al. 2022; Sivakanthan et al. 2022). However, high consumption of solid fats increases the risk of cardiovascular diseases, obesity, and diabetes due to the saturated fatty acids they contain (Pehlivanoğlu et al. 2018). Due to health and economic concerns, solid fats have increasingly been replaced by structured liquid vegetable oils.

Oleogels derived from oils represent an innovative approach that seeks to mitigate the adverse health effects of oils while providing the structural benefits of fats (Singh et al. 2017). Oleogelation is an innovative technique in which large amounts of liquid oil are thermally reversible and retained in a three‐dimensional gel matrix, forming solid‐like structured materials (Demirkesen and Mert 2020). Since oleogels can act as barriers against oxygen, free radicals, and light, they effectively protect bioactive compounds from degradation and enhance their bioavailability (Ramezani et al. 2024). Upon examining the studies, it is evident that oleogels have been successfully utilized in various food products such as cheese (Moon et al. 2021), ice cream (Jing et al. 2022), sponge cake (Chen et al. 2023), croissants (Espert et al. 2023), and sausage (Igenbayev et al. 2023).

The type of oil used in oleogel production is a critical factor that determines the final properties of the oleogel. The oil's chemical structure, the amount of unsaturated fatty acids, the length of the fatty acid chains, and their polarity all influence the structural integrity of the oleogel (Manzoor et al. 2022). The proper selection of oil for oleogel formulation helps preserve foods' desired sensory and physical properties. It provides a healthy alternative due to its unsaturated fatty acids, antioxidant content, and phenolic compounds. EVOO, which plays a significant role in the Mediterranean diet and is used in this study, comprises 98–99% fatty acids (primarily monounsaturated fatty acids, such as oleic acid) and 1–2% of bioactive compounds, including phenolics, phytosterols, tocopherols, and squalene. Thanks to these components, EVOO is a lipid source recognized for its beneficial effects, particularly in preventing cardiovascular diseases (Ciuffarin et al. 2023; Romani et al. 2019).

Another critical factor influencing oleogel production is the choice of oleogelators used. The most effective oleogelators have been reported to be waxes due to their ability to crystallize even at low concentrations. Natural waxes commonly used in oleogel production include beeswax, sunflower, rice bran, carnauba, and CDW. CDW has a relatively low melting point (70.5°C) compared to most other waxes. It is also approved by the FDA as a food additive, with no restrictions on its use (Yilmaz et al. 2021).

One of the key applications of oils is frying, a process in which foods are immersed in hot oil (150‐190°C) to cook them (Aydeniz Guneser et al. 2021). The thermal behavior and frying performance of olive oil have been extensively studied. Heating EVOO increases PV and decreases phenolic content, although it remains more stable than many refined oils (Giuffrè et al. 2018). Prolonged heating further accelerates the formation of polar compounds and FFA (Giuffrè et al. 2017). During deep‐frying, olive oil still undergoes degradation over multiple frying cycles (Khattab 2022). To address these limitations, structured systems like oleogels have been proposed to improve frying performance.

Although frying yields desirable texture, aroma, and color, it also promotes oxidation, hydrolysis, and polymerization reactions, leading to the formation of harmful compounds (Thakur et al. 2023). In parallel, oils are absorbed into the food matrix. Excessive oil intake is linked to chronic diseases, prompting interest in reducing oil absorption while maintaining quality (Mahmud et al. 2023). Recent studies suggest that oleogels can reduce oil uptake in fried foods (Adrah et al. 2022; Çakır et al. 2023).

Moreover, recent research has concentrated on the behavior of oleogels during the process of digestion. In vitro studies have demonstrated that oleogel structure may reduce lipolysis and delay free fatty acid release, thereby potentially influencing energy absorption and bioactive compound bio accessibility (Ciuffarin et al. 2023; Lu et al. 2023). These findings lend support to the exploration of oleogels as not only functional fat replacers but also nutritionally beneficial systems.

The aim of this study is to (i) investigate the suitability of an oleogel obtained from EVOO for deep frying; (ii) xamine the storage stability of the obtained oleogel under different storage conditions and compare it with oil; and (iii) monitor the bio accessibility of bioactive components in EVOO and the oleogel derived from this oil after in vitro digestion.

## 2 Materials and Methods

### 2.1 Materials

EVOO (Tariş North Aegean EVOO, Izmir, Türkiye) was purchased from a local market. CDW was obtained from Ravago Petrochemicals (Istanbul, Türkiye). Isooctane, p‐AV, glacial acetic acid, chloroform, potassium iodide, starch, sodium thiosulfate, KCl, KH₂PO₄, NaHCO₃, MgCl₂·6H₂O, pepsin, CaCl₂, NaCl, NaOH, (NH₄)₂CO₃, pancreatin, α‐amylase, and bile salts were purchased from Sigma‐Aldrich Chemie GmbH (Germany). All chemicals were of analytical grade. Potatoes for frying were purchased from a local market.

### 2.2 Preparation for Oleogel

Oleogels were prepared using the method described by (Zubairee et al. 2024)., by mixing 24.25 g of EVOO with 0.75 g of CDW (3%, w/w) in a beaker using a magnetic stirrer (Daihan Scientific, SMHS‐3, South Korea) at 700 rpm. The mixture was heated to 75°C to melt the wax. Once the wax was completely melted and a homogeneous mixture was achieved, it was cooled to room temperature for approximately 10 min and stored at 4°C for 24 h.

### 2.3 Analysis of Oils and Oleogels

#### 2.3.1 OBC

Oleogels were melted in a water bath at 90°C to measure OBC. Approximately 1 mL of the sample was transferred into pre‐weighed Eppendorf tubes (*a*). The tubes were then kept at 4°C for 1 h for re‐gelling. After gel formation, the re‐weighed tube; and (*b*) were centrifuged (Centrifuge Hettich, Germany) at 20°C and 10,000 rpm for 15 min. Following centrifugation, the samples were inverted and rested for 3 min before being re‐weighed (*c*). The %RBV was calculated using the following Equation (1):

(1) $$\begin{array}{c} \%\;\mathrm{Free}\;\mathrm{Fat}=\left(\left(b-a\right)-\left(c-a\right)\right)/\left(b-a\right)\times 100\\ \%\;\mathrm{OBC}=100-\%\;\mathrm{Free}\;\mathrm{Fat} \end{array}$$

#### 2.3.2 Gelation Time

The test tubes containing oleogel were fully melted in a water bath at 90°C and maintained for two hours to stabilize the temperature. Then, they were brought to room temperature, and the time required for complete gelation was determined. The crystallization time was recorded as the point at which flow ceased when the tubes were tilted to a 90° angle, as described by (Dassanayake et al. 2009).

#### 2.3.3 Rheological Analysis of Oleogel Samples

The rheological properties of the oleogel samples were measured using a rheometry device (ThermoHAAKE, Mars III, Karlsruhe, Germany). For this, parallel plate geometry (gap 1 mm, diameter 35 mm) was used. Approximately 1 g of the oleogel sample was placed between two flat parallel plates, and rheological measurements were performed at 25°C over a frequency range of 1–100 s⁻¹, generating 25 data points. The data were analyzed using RheoWin Data Pro software. A frequency sweep test was conducted to evaluate the viscoelastic properties of the oleogel samples. The storage modulus (G′), loss modulus (G″), and tan(δ) (G″/G′) values were measured over a frequency range of 0.1–10 Hz under a constant stress of 0.2 Pa.

#### 2.3.4 Smoke Point

The smoke point of the oil and oleogel was determined by heating 100 mL of the sample in a beaker until a continuous bluish smoke was observed. The temperature at this point was recorded using a digital thermometer (Chauhan et al. 2022).

### 2.4 Deep‐Fat Frying

A deep fryer (Korkmaz A486‐02 Vertex 900 W, Istanbul, Türkiye) was used for frying. The potato slices used in the frying process were weighed ∼100 g for each frying process and fried for 3 min in oil‐oleogel, reaching 180°C. This process was repeated 20 times without adding fresh oil. After the 5th, 10th, 15th, and 20th frying, samples of oil‐oleogel and potatoes were analyzed.

#### 2.4.1 Oil Uptake

The total fat content of the fried potatoes was determined using a Soxhlet extraction apparatus, following the method described by Dursun Capar and Yalcin (2017). Approximately 6–8 grams of potato sample was used, and the fat was extracted using hexane as the solvent. The fat content was then calculated according to the procedure outlined in the referenced method.

#### 2.4.2 Color Analysis for Potatoes

The color parameters of the deep‐fried potatoes, including the L* value (where L = 0 is equivalent to black and L = 100 is comparable to white) and the a* and b* values (–a indicates greenness, + a indicates redness; –b indicates blueness, + b indicates yellowness), were measured using a Konica Minolta Chroma Meter (CR‐5, Osaka, Japan). Before measurement, the device was calibrated using a white calibration plate. After the fifth, tenth, fifteenth, and twentieth frying processes, measurements were performed in triplicate for each sample, with five repetitions per measurement, and the average values were recorded.

#### 2.4.3 FFA Analysis

The IUPAC method determined the free fatty acidity in fried oleogel‐oil samples (Anonymous 1987). A 5–10 g sample was weighed and dissolved in 50–100 ml of an ethanol‐diethyl ether solution (1:1, v/v) by shaking for one minute. The solution was titrated with a 0.1 *N* ethanol potassium hydroxide solution until a pink color was formed. The FFA was calculated according to Equation (2). The total amount of unbound fatty acids in the oils is expressed as a percentage of oleic acid.

(2) $$\%\;\mathrm{Free}\;\mathrm{fatty}\;\mathrm{acidity}:\;\left(V\times T\times Ma\right)/m$$

Where *V* is the volume of potassium hydroxide solution with ethanol (mL), *T* is the normality of the potassium hydroxide solution with ethanol, *Ma* is the molecular weight of oleic acid, and *m* is the mass of the sample (g).

#### 2.4.4 PV Determination

The PV was determined using the AOACS method (AOAC 2000). The samples to be analyzed were weighed 2 g into the flasks, 10 ml of chloroform was added, and the contents were shaken rapidly to facilitate the dissolution of the oil. Subsequently, 15 ml of acetic acid and 1 ml of a KI (potassium iodide) solution were added, shaken for an additional minute, and then incubated in the dark for 10 min. Subsequently, 75 ml of distilled water and 1 ml of starch solution were added, and the sample was titrated with 0.01 *N* sodium thiosulfate. The quantity of thiosulfate consumed was then recorded. The peroxide values were calculated following Equation (3):

(3) PV=V×T×1000/mmeqgO2/kg

Where *V* is the volume of sodium thiosulfate used in the titration (mL), *T* is the normality of the sodium thiosulfate solution, and *m* is the mass of the sample (g).

#### 2.4.5 p‐AV Value Determination

The AOACS method was employed to ascertain the p‐AV value of oleogel‐oil samples obtained following frying (AOAC 1998). A 0.5 g sample was dissolved in 25 mL of isooctane. Subsequently, 5 mL of this mixture was transferred into a flask, and 1 mL of p‐AV solution was added. The solution was incubated in the dark for 10 min, after which the absorbance was measured at 350 nm against the isooctane solution using a spectrophotometer (UV 160A, Shimadzu, Japan). The p‐AV value was calculated using Equation (4):

(4) $$p-\mathrm{AV}:25\times 1.2\times \left(A_{2}-A_{1}\right)/m$$

Where *A_1_* is the absorbance value of the solution prepared with isooctane, *A_2_* is the absorbance value of the solution prepared with p‐AV, and *m* is the weight of the sample used for the analysis.

### 2.5 Storage Stability

To examine the storage stability, oleogel and EVOO samples were stored at room temperature for 90 days, one group in a closed cabinet with no light and one in an open environment exposed to daylight during the day. On days 0, 30, 60, and 90, the OBC, FT‐IR, XRD, p‐AV, FFA, and PV analyses were performed.

#### 2.5.1 OBC

The OBC of the stored oleogels was determined by the methodology outlined in Section 2.3.1.

#### 2.5.2 XRD

A Cu radiation source (λ = 1.54056 Å) was used in the diffract meter to investigate the diffraction patterns of the stored oleogels. The analysis was conducted under the following conditions: a voltage of 40 kV, a current of 40 mA, a step size of 0.02°, and a scanning speed of 2°/min. The diffraction curve was recorded within a diffraction angle (2θ) range of 2° to 50°.

#### 2.5.3 FTIR Spectra

The FTIR spectra of the stored oleogels were analyzed using an FTIR spectrophotometer (Spectrum 400, Perkin Elmer Instruments, Waltham, USA). The samples were scanned within the range of 400–4000 cm⁻¹.

#### 2.5.4 Oxidative Stability Analyses

The oxidative stability of the stored samples was evaluated through the analysis of FFA, PV, and p‐AV. The FFA values were determined by the methodology described in Section .4.3, the PV according to Section 2.4.4, and the p‐AV values outlined in Section 2.4.5.

### 2.6 In Vitro Digestion Model

The gastrointestinal tract was simulated using the INFOGEST method (Minekus et al. 2014) to investigate the behavior of oleogel during digestion and compare it with the digestion of oil. The guidelines provided by Sabet et al. (2022) were also considered when conducting this study.

#### 2.6.1 Oral Phase

In the oral phase, 0.258 g of oleogel (3% wax) and 0.25 g of the oil samples were weighed and adjusted to 5 g by adding distilled water. Subsequently, 4 mL of SSF, preheated to 37°C, 0.975 mL of distilled water, and 25 µL of CaCl₂ were added. The mixture was then placed in a shaking water bath (model BS‐21, manufactured in Korea) at 37°C for 2 mins.

#### 2.6.2 Gastric Phase

To the oral bolus, 8 mL of SGF, preheated to 37°C, was added, followed by pH adjustment to 3 using HCl. Subsequently, 0.5 mL of pepsin, five µl of CaCl₂, and distilled water (the total volume of HCl, CaCl₂, and water used for pH adjustment was 1 mL) were introduced. The resulting mixture was then incubated in a shaking water bath at 37°C for 2 h.

#### 2.6.3 Intestinal Phase

Subsequently, 8.5 mL of SIF was preheated to 37°C and added to the gastric chymus. The pH was then adjusted to seven using NaOH. Following this, 40 µL of CaCl₂, 5 mL of pancreatin, 2.5 mL of bile salts, and distilled water (the total volume of NaOH, CaCl₂, and distilled water used for pH adjustment was 4 mL were introduced. The resultant mixture was then incubated in a shaking water bath at 37°C for 2 h.

The digestive fluid obtained after the analysis was subjected to centrifugation at 9,000 rpm and 4°C for 30 min to prepare for subsequent analyses.

#### 2.6.4 Lipolysis

The lipid digestion rate was calculated by carefully documenting the NaOH used to neutralize the FFAs produced during intestinal lipolysis. Equation (5) was employed for this calculation (Ashkar et al. 2019).

(5) $$\%\mathrm{FFA}=100\times \left(\left(V_{NaOH}\times M_{NaOH}\times M_{lipid}\right)/\left(2\times W_{lipid}\right)\right)$$

Where *V_NaOH_*, *M_NaOH_*, *M_Lipid_*, and *W_Lipid_* refer to the volume of NaOH used (mL), the molarity of NaOH (M), the molecular weight of extra virgin olive oil (g/mol), and the total amount of oil initially present (g), respectively.

#### 2.6.5 Tocopherol Bioaccessibility

Following in vitro digestion, 3 ml of the resulting digestive fluid (digesta) and the micellar phase of the centrifugally concentrated liquid post‐digestion were collected. These were then mixed with hexane/ethanol (1:1, v/v) and subjected to centrifugation at 4,000 rpm for 2 min. This process was repeated three times to obtain the upper liquid layer. The upper liquid layers were combined, and the α‐tocopherol concentrations were determined through HPLC analysis. Agilent 1260 Series high‐pressure liquid chromatography (HPLC), 1260 G uat pump, and a maximum injection loop volume of 500 µL were used (Lv et al. 2019). The bio accessibility of α‐tocopherol was calculated using Equation (6):

(6) $$\mathrm{Bio}-\mathrm{accessible}\;\alpha -\mathrm{tocopherol}\;\left(\%\right)=C_{micella}/C_{digesta}\times 100$$

In this experiment, *C_misella_* indicates the amount of tocopherol in the micellar fraction, and *C_digesta_* suggests the amount of α‐tocopherol in the digestive fluid. Stability was calculated as the percentage of α‐tocopherol concentration in the digesta that remained untransformed regarding the initial α‐tocopherol concentration (*C* = 22.47 mg/l for EVOO and 10.77 mg/l for oleogel), according to Equation (7):

(7) $$\mathrm{Stability}\;\left(\%\right)=\left(C_{digesta}/C\right)\times 100$$

Bioavailability estimates the degree of α‐tocopherol absorption by considering the product of the stable and bioaccessible bioactive compounds Equation (8) (Fernandes et al. 2024).

(8) $$\mathrm{Bioavailability}=\left(\mathrm{Bioaccessibility}\times \mathrm{Stability}\right)\times 100$$

#### 2.6.6 Β‐Carotene Bioaccessibility

The β‐carotene content in the digestion product was determined by vortexing 0.5 mL of digesta, taken after the small intestine phase, with 5 mL of hexane‐ethanol (3:2) at 25°C for 1 min. The resulting upper layer was then analyzed using a spectrophotometer (UV 160A, Shimadzu, Japan) at a wavelength of 450 nm. To determine the amount of β‐carotene in the micellar phase, 0.5 mL of the sample was subjected to centrifugation (9,000 rpm at 4°C for 30 min) after the small intestine phase and subsequently analyzed according to the established methodology. A hexane‐based standard curve quantified the β‐carotene content in the digestive fluid and micellar phase. The amount of bio accessible β‐carotene was calculated using Equation (9), as Ramezani et al. (2024) outlined.

(9) $$\mathrm{Bio}-\mathrm{accessible}\;\beta -\mathrm{carotene}\;\left(\%\right)=C_{misella}/C_{digesta}\times 100$$

Where *C_micella_* represents the β‐carotene content (mg/g oleogel‐oil) in the micellar fraction, and *C_digesta_* represents the β‐carotene content in the digestive fluid.

Equation (10) was used to calculate the stability and bioavailability of β‐carotene (*C* = 1.06 mg/l for EVOO and 0.92 mg/l for oleogel).

(10) $$\begin{array}{c} \mathrm{Stability}\;\left(\%\right)=\left(C_{digesta}/C\right)\times 100\\ \mathrm{Bioavailability}=\left(\mathrm{Bioaccessibility}\times \mathrm{Stability}\right)\times 100 \end{array}$$

### 2.7 Statistical Analysis

The experiments were conducted in triplicate. The significance of the results was analyzed using IBM SPSS 26 statistical software. Pairwise comparisons were performed using the independent t‐test. Multiple comparisons were assessed using analysis of variance (ANOVA) followed by Duncan's multiple range test (*p* < 0.05).

## 3 Results and Discussions

### 3.1 Analysis of Oils and Oleogels

#### 3.1.1 OBC

The OBC is a critical quality parameter for oleogels due to their high oil content. Accordingly, oleogels are expected to exhibit elevated OBC values (Thakur et al. 2022). The oleogel used in this study consists of 97% oil by weight, with an OBC value exceeding 99%. This high OBC value demonstrates the oleogel's strong ability to retain oil within its structure (Zubairee et al. 2024). The elevated OBC may be attributed to the type of oil and oleogelators used. Sivakanthan et al. (2024) reported that oleogels prepared from untreated oils exhibit high OBC. Additionally, oleogels incorporating CDW have been shown to retain more significant amounts of liquid oil within their crystalline structure. This property can be attributed to the wax's high surface area and extensive oil phase dispersion. (Doan et al. 2018).

#### 3.1.2 Gelation Time

Gelation time in oleogels refers to the period required for liquid oils to transition into a gel‐like structure. Several factors, including gelator concentration, cooling rate, and storage conditions, influence this parameter (Keskin Uslu and Yılmaz 2021). In commercial production, shorter gelation times are beneficial (Yilmaz et al. 2021). Previous studies have demonstrated that gelation time increases as gelator concentration decreases (Ash et al. 2021). However, in the present study, even at a low gelator concentration of 3%, the gelation time of the oleogel was determined to be relatively short at 8.07 min. This finding aligns with the results of previous studies (Öğütcü et al. 2015; Öğütcü and Yılmaz 2015).

#### 3.1.3 Rheological Properties of Oleogel

**FIGURE 1:**
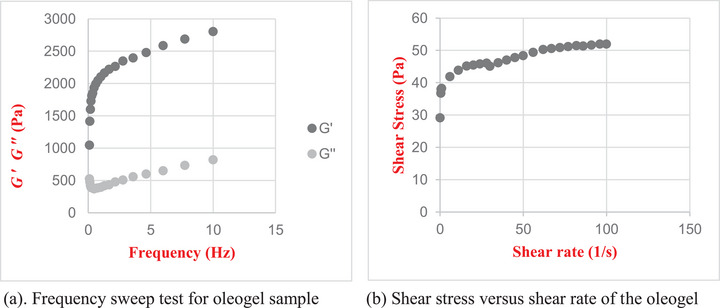
(a) Frequency sweep test for oleogel sample; and (b) Shear stress versus shear rate of the oleogel.

Rheological analyses are a means of obtaining information regarding the structural integrity of products. Steady‐state analyses are commonly performed to examine the flow behavior of gels. At the same time, oscillatory tests are used to distinguish between viscous sol (G' < G″) and gel (G' > G″) states (S. Yang et al. 2024). The structural characteristics of gels can be classified into three distinct categories: strong gels, defined by a G″/G′ ratio of ≤ 0.1; weak gels, with a ratio of 0.1< G″/G′ < 1; and viscous sols, where the ratio is G″/G′ ≥ 1 (Zubairee et al. 2024). As depicted in Figure 1. a, the variation in G' (storage modulus) and G″ (loss modulus) values is evident within the frequency range of 0.1–10 Hz for the oleogel samples. The storage modulus (G') and loss modulus (G″) values increased concurrently as the frequency increased. This observation suggests that the network structure of the oleogels was likely formed through non‐covalent interactions (Farooq et al. 2023). In instances where the G' value exceeds the G″ value, it is widely accepted that a gel structure has been formed. Figure 1. a demonstrates that the G' value consistently exceeds the G″ value across all frequency ranges. This observation confirms that the oleogel derived from EVOO and CDW exhibits a gel structure, as the storage modulus (G′) was consistently higher than the loss modulus (G″) across all tested frequencies.

As demonstrated in Figure 1.b, the steady shear rheological measurement values for the oleogels are presented. The oleogel samples exhibited a pronounced shear‐thinning behavior. The analysis of the relationship between shear stress and shear rate, as shown in Figure 1.b, revealed a high *R* ^2^ value (0.97–0.99), consistent with findings reported by (Kwon and Chang 2022). The flow behavior index (*n*) value of the oleogel was found to be 0.10. When the behavior index of the fluid is less than one, it exhibits shear‐thinning behavior, and as the index approaches one, the behavior of the fluid transitions from shear thinning to shear thickening. Conversely, when the index is greater than one, the fluid exhibits shear‐thickening behavior (Li et al. 2024; Zubairee et al. 2024). Zubairee et al. (2024) determined the flow behavior index of oleogel synthesized using sunflower oil and soybean wax to be 0.52. In a separate study, Kwon and Chang (2022) reported the *n*‐index values of oleogels to range from 0.52 to 0.63. The parameter *K*, known as the consistency index, estimates viscosity. The numerical value of *K* was found to be 36.96, while the apparent viscosity (η) was determined to be 0.97. (Zubairee et al. 2024) reported *K* and η values of 1.263 and 0.18, respectively.

A decrease in the *n* value and an increase in the *K* and η values in oleogels may indicate the formation of stronger intermolecular networks within the gel structure (Kwon and Chang 2022). The lower *n* and higher *K* and η values indicate the presence of stronger intermolecular interactions, which supports the hypothesis that a more rigid gel matrix is present in the formulation.

These rheological characteristics, particularly the dominance of elastic behavior (G′ > G″) and low flow behavior index (*n*), are consistent with the structurally stable gel network supported by the FTIR spectra and the presence of β′‐type crystalline structures observed in the XRD pattern.

#### 3.1.4 Smoke Point

The smoke point of an oil is defined as the temperature at which the oil begins to decompose into FFA and glycerol, producing continuous bluish smoke. The formation of acrolein, a compound with a characteristic odor and taste, is a consequence of this process (Thakur et al. 2022). The smoke point of EVOO was determined to be 187°C, while that of oleogel was found to be 205°C. The study revealed that incorporating CDW led to an enhancement in the smoke point. Using oleogelators has enhanced the smoke point in previous studies (Chauhan et al. 2022; Thakur et al. 2022). Moreover, the utilization of gelators has been demonstrated to enhance the oxidative and thermal stability of oils. The gel matrix formed has been shown to reduce the availability of free fat molecules, thereby reducing the tendency to break down and smoke at lower temperatures (Dimakopoulou‐Papazoglou et al. 2024).

### 3.2 Deep‐Fat Frying

#### 3.2.1 Oil Uptake and Color Analysis for Potatoes

Oil absorption during frying is critical in determining foodstuffs' nutritional value and energy content. Consumers of high‐fat foods are at an increased risk of developing health issues, including cardiovascular diseases, obesity, and other metabolic disorders (Mahmud et al. 2023). Consequently, reducing the rate of fat absorption during frying decreases the calorie content of the food, thereby contributing to the production of healthier products. The oil absorption rates of oil‐ and oleogel‐fried potatoes were calculated as 14.51% and 10.29%, respectively. The oil absorption of products fried in oleogel was significantly lower (*p* < 0.05) than that of products fried in oil (Table 1). Chauhan et al. (2022) reported that the oleogel structure's wax adheres to the fried product's surface, forming a coating that reduces water migration and, consequently, oil absorption. In addition to the physical barrier formed by the wax on the product surface, the high OBC of the oleogel (99.96%) likely contributed to the reduction in oil absorption by limiting the amount of free oil available during frying. In a study, mathri, an traditional Indian product, was fried in refined soybean oil and oleogels containing different concentrations of wax. The fat content of products fried in oil was 27.14%, while the fat content of products fried in oleogels with 5%, 10%, and 15% wax was 19.61%, 21.05%, and 21.89%, respectively.

**TABLE 1:** Oil absorption and color characteristics of fried potatoes.

Frying environment	Oil uptake (%)	L*	a*	b*
Oleogel	10.29 ± 0.74^B^	66.29 ± 0.03^A^	−1.45 ± 0.04^B^	25.03 ± 0.03^B^
EVOO	14.51 ± 0.93^A^	65.10 ± 0.26^B^	3.41 ± 0.13^A^	32.06 ± 0.42^A^

*Note*: Mean values with same letters in the same column are not statistically significant (*p* > 0.05). EVOO: Extra virgin olive oil.

Furthermore, it was observed that the oil absorption rate decreased as the wax concentration in oleogels increased (Chauhan et al. 2022). In their study, Zubairee et al. (2024) examined the oil uptake of fried donuts using oleogel and sunflower oil as frying media. The results indicated that donuts fried in oleogel absorbed 37.8% less oil than those fried in sunflower oil. The results demonstrate that oleogel effectively reduces oil absorption when used as a frying medium in the deep‐fat frying process. The EVOO–CDW oleogel significantly reduced oil absorption in fried potatoes (*p* < 0.05), confirming its potential to produce lower‐calorie fried products. This finding aligns with prior reports and underscores its industrial relevance for developing healthier fried foods.

The color of fried potatoes results from Maillard and non‐enzymatic browning reactions, which depend on the reducing sugar content on the surface, temperature, and frying time (Pathare et al. 2013). The desired color of fried potatoes is a bright golden yellow. Table 1 presents the color values of potatoes fried in oil and oleogel. The L (brightness) values of the fried potatoes showed that those fried in oleogel were brighter than those fried in oil. This outcome is consistent with the findings of earlier research. Using oleogels in frying reduces oil absorption and enhances the fried products' sensory appeal, making them more visually appealing and potentially palatable. (Aydeniz Guneser et al. 2021; Zubairee et al. 2024).

The a* value of fried potatoes in oleogel was negative, indicating that the samples exhibited green tones. The b* values of the samples, which were positive, showed a yellow color. The b* value of the samples fried in the EVOO medium was higher. It was reported that the b* value was higher in samples with higher oil absorption rates (Aydeniz Guneser et al. 2021). This result is consistent with the findings of our study.

#### 3.2.2 Chemical Properties of Oleogel

FFA are essential indicators of oils' hydrolytic and oxidative degradation processes. Oils exposed to elevated temperatures during frying can break down into fatty acids, adversely affecting their quality. Table 2. shows the FFA values of frying oils. After 60 min of frying at 180°C, corresponding to the final 20 min, no significant difference was observed in the FFA levels between the oleogel and oil samples (*p* > 0.05). At the beginning, the FFA values of oleogel and oil were 0.87 ± 0.07 and 0.78 ± 0.08, respectively, while at the end of the 20th frying cycle, they were 1.05 ± 0.06 and 0.92 ± 0.18, respectively. The permissible FFA levels in some European countries range from 0.9% to 2.5% (Hosseini et al. 2016). At the end of the 20th frying cycle, it was observed that these values were not exceeded in either oleogel or oil. The oleogel, used as a frying medium, was found to be suitable for frying in terms of its FFA value.

**TABLE 2:** FFA, peroxide, and p‐AVvalue changes in oleogel and EVOO during frying.

Property	Sample	Number of frying				
		0	5	10	15	20
FFA (%)	Oleogel	0.87 ± 0.07^A^	0.94 ± 0.12^A^	0.98 ± 0.09^A^	1.04 ± 0.05^A^	1.05 ± 0.06^A^
	EVOO	0.78 ± 0.08^A^	0.79 ± 0.08^A^	0.82 ± 0.01^B^	0.84 ± 0.00^B^	0.92 ± 0.18^A^
PV (meq O_2_ kg^−1^)	Oleogel	0.62 ± 0.31^A^	4.43 ± 0.78^A^	4.79 ± 0.16^B^	5.43 ± 0.75^B^	4.62 ± 0.49^B^
	EVOO	0.48 ± 0.21^A^	6.70 ± 1.40^A^	6.88 ± 0.41^A^	7.90 ± 0.93^A^	9.22 ± 0.48^A^
p‐AV	Oleogel	1.64 ± 0.09^A^	16.64 ± 0.24^A^	17.14± 1.00^A^	17.20 ± 0.70^A^	17.40 ± 0.53^B^
	EVOO	0.55 ± 0.38^B^	15.75 ± 0.83^A^	16.23 ± 0.40^A^	17.27 ± 0.11^A^	19.68 ± 1.10^A^

*Note*: Mean values with same letters in the same column are not statistically significant (*p* > 0.05). EVOO: Extra virgin olive oil.

Oil oxidation occurs due to a series of chemical reactions triggered by exposure to elevated temperatures and oxygen, adversely affecting the quality of the oil. PV is one of the most important parameters used to monitor the oxidative stability of oil. The upper limit of the PV value is < 15–20 meq kg⁻¹. A PV value greater than 20 meq kg⁻¹ indicates significant off‐flavors and poor quality (Thakur et al. 2023). As demonstrated in Table 2, the PV of oleogel and EVOO were 4.62 ± 0.49 and 9.22 ± 0.48, respectively, at the end of the 20th day. These values are below the upper limits. Until the 10th frying, there was no significant difference between the PV of oleogel and oil. However, after the 10th frying, the PV of oleogel were statistically lower than those of EVOO. This may be explained by the fact that the solid structure of oleogel can potentially delay the passage of oxygen into the oil, especially when it re‐solidifies at room temperature after frying (Aydeniz Guneser et al. 2021). Oleogels were found to be more stable against oxidation compared to EVOO. This finding is in agreement with the higher smoke point observed for oleogel, suggesting that the gel structure provides thermal protection that may also contribute to oxidative stability during frying. This result is consistent with other studies in the literature (Aydeniz Guneser et al. 2021; Lim et al. 2017; Zubairee et al. 2024). These findings indicate that oleogel is a suitable alternative to oil as a frying medium.

The p‐AV is an essential parameter for assessing oils' advanced stages of oxidative degradation. It precisely measures the degradation of peroxides, the primary oxidation products, into secondary oxidation products such as aldehydes. As demonstrated in Table 2, no statistically significant difference was observed between the p‐AV values of oleogel and EVOO after the 5th, 10th, and 15th frying. At the end of the 20th frying, the p‐AV values of oleogel samples were lower than those of EVOO (*p* < 0.05). This result indicates that oleogel is more stable against secondary oxidation than EVOO during the progressive frying process. The present results are consistent with those of previous studies. In a study where oleogels obtained from beeswax‐grape seed oil and carnauba wax‐rapeseed oil were subjected to frying, the p‐AV value was found to be higher in the oil samples than in the oleogels (Yi et al. 2017). In a separate study, sunflower oil oleogel was produced using rice wax, and frying with sunflower oil was used as a control group. The results showed that p‐AV values were lower in oleogel compared to sunflower oil (Tajer and Ozdemir 2023). Similarly, another study observed that oleogel used as a frying medium had lower p‐AV values than oil (Zubairee et al. 2024). According to the results of the present study and similar findings in the literature, oleogels reduce oil oxidative deterioration in the frying environment. This effect is believed to be due to the gelator, which reduces the direct contact of oil molecules with oxygen, thereby slowing down oxidative degradation.

When considering the chemical analysis results of oleogel and EVOO used as frying media, it is evident that oleogel exhibits more excellent stability against oxidation. Therefore, oleogel derived from EVOO and CDW can be considered a promising alternative to oil as a frying medium, with the added industrial advantage of potentially extending the usable life of frying oils, reducing waste, and maintaining product quality over multiple frying cycles.

### 3.3 Storage Stability

#### 3.3.1 OBC

**FIGURE 2:**
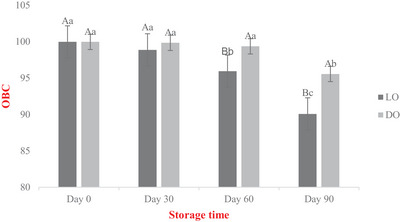
OBC values of oleogels during storage. Mean values of the same capital letters are not statistically significant among applications (*p* > 0.05). Mean values of the same small letters are not statistically significant during storage (*p* > 0.05). LO: Light‐stored oleogel, DO: Dark‐stored oleogel.

The changes in the OBC value of oleogel during storage were calculated on days 0, 30, 60, and 90, and the results are given in Figure 2. The OBC value of oleogels stored in light and dark conditions, which was 99.96% at the beginning, decreased in the following days of storage. The OBC value of oleogel stored under dark conditions (DO) was observed on day 90, while the OBC value of oleogel stored under light conditions (LO) was observed on day 60. Until the 60th day, no significant difference in the OBC value of both oleogel samples was observed (*p* > 0.05), while the OBC value of LO was found to be lower on the 60th day and after (*p* < 0.05). Considering these results, it was observed that light hurt OBC.

#### 3.3.2 XRD

**FIGURE 3:**
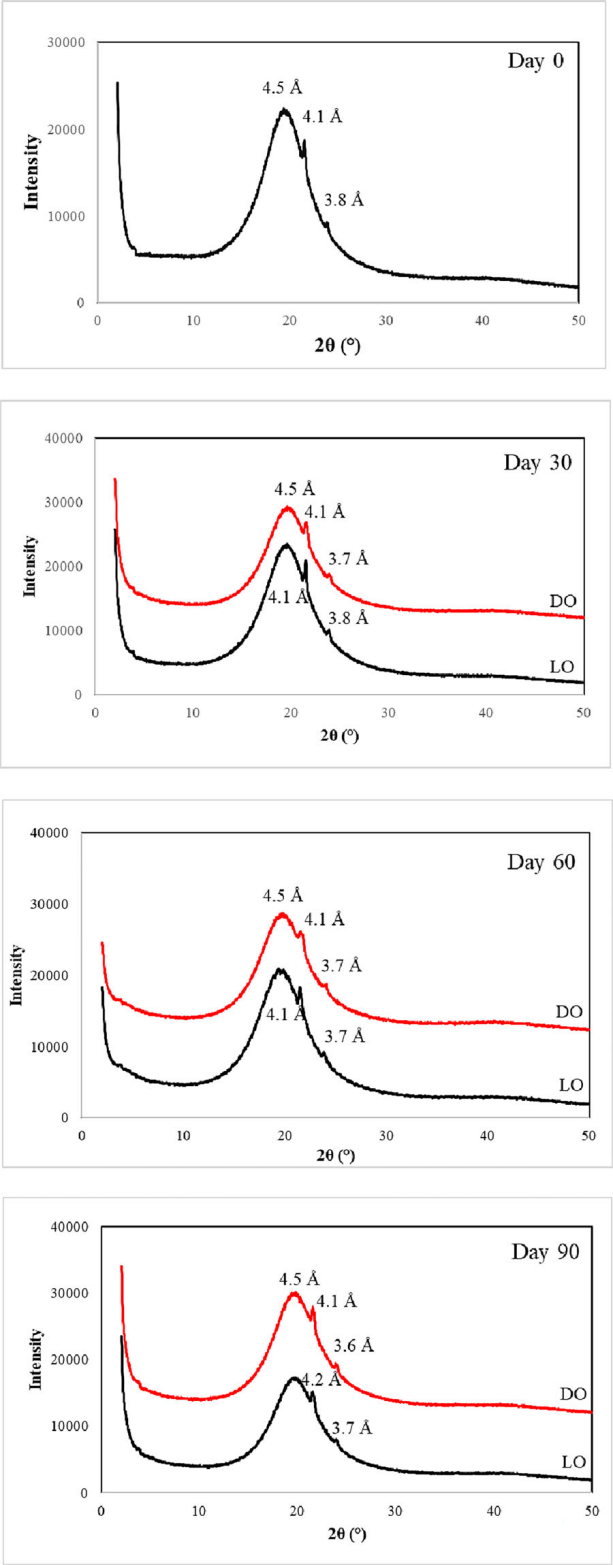
X‐ray diffraction pattern of oleogels during storage. LO: Light‐stored oleogel, DO: Dark‐stored oleogel.

XRD patterns of oleogels during storage are presented in Figure 3. The oleogel displays diffraction peaks at d = 4.5 Å, d = 4.1 Å, and d = 3.8 Å. The three most frequently observed polymorphic structures in oils are α, β′, and β, in order of increasing stability (Chaturvedi et al. 2023). The presence of two diffraction peaks at 4.1 Å and 3.8 Å corresponds to orthorhombic perpendicular subcellular packing arrangements. These peaks are similar to those of β′ type triacylglycerol. Due to the small size of the crystals in this form of fat, it exhibits a smooth texture with high spread ability, making it the most popular choice in the food industry, particularly in the production of margarine and solid fats (Ghazani et al. 2022; Zhou et al. 2025). The diffraction peak at d = 4.5 Å indicates the characteristic peak of β crystals in wax crystals (J. Yang et al. 2022). Similar characteristic diffraction peaks have been observed in gels obtained using gelling agents such as rice bran wax and beeswax (Ghazani et al. 2022; J. Yang et al. 2022). It was observed that 90 days of storage and light had no significant effect on the XDR, indicating that the crystalline structure of the oleogel remained stable over time and was not altered by environmental conditions.

#### 3.3.3 FTIR Spectra Analysis

**FIGURE 4:**
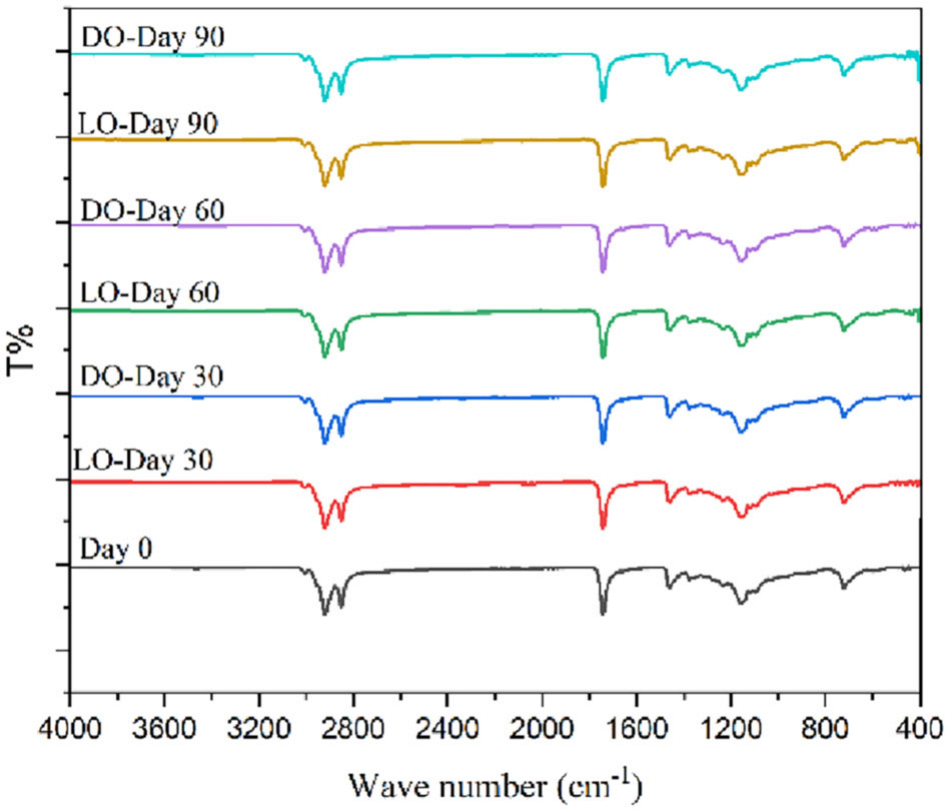
FTIR spectra of oleogel samples.

FTIR spectroscopy is a widely utilized analytical technique for characterizing molecular structures. This study obtained FTIR spectra to investigate the chemical bond interactions within the oleogel samples and monitor their variations throughout storage. The corresponding results are presented in Figure 4. No significant differences were observed in the spectra of oleogels during the storage period. The peaks observed at 3002–3007 cm^−1^ correspond to the stretching vibrations of CH bonds, which are indicative of the polyunsaturation of the oil. Peaks at 2920 and 2851 cm^−1^ indicate CH stretching of CH_3_ and CH_2_ in EVOO and CDW, while CH bending of CH_3_ and CH_2_ appeared at 1457 cm^−1^ and 1357 cm^−1^, respectively (Liu et al. 2021). The peaks observed at 1742 cm^−1^ can be attributed to the C = O stretching of esters and FFA, while those at 1157–1158 cm^−1^ correspond to the stretching of C‐O bonds of aliphatic esters and CH_2_ bending vibrations (Barragán‐Martínez et al. 2022). The peaks at approximately 2920 cm^−1^, 2851 cm^−1^, and 1742 cm^−1^ indicate the abundance of triglycerides in the oleogel. The peak at approximately 720 cm^−1^ can be attributed to the stretching vibration of the CH_2_ group, which forms the long‐chain mono‐ and polyunsaturated fatty acids present in the oil (Pușcaș et al. 2020).

The absence of new peaks or shifts in the FTIR spectra throughout the storage period suggests that no chemical reactions or molecular interactions took place in the oleogel structure, thereby supporting its chemical stability during storage.

#### 3.3.4 Chemical Properties of Oleogel and Oil

FFA, an important parameter for evaluating oxidative stability and quality changes of oils, was assessed on the designated analysis days, and the results are presented in Table 3. Following the conclusion of the storage period, a significant increase in the FFA value was observed in all samples compared to their initial values (*p* < 0.05). The lowest increase was observed in oil samples stored in the dark (DOO), while the highest increase was observed in oleogel samples stored in both light and dark. Based on these results, it can be stated that oleogels exhibited lower stability in terms of FFA formation compared to oil. When the oil samples were compared within themselves, those exposed to daylight exhibited higher FFA values. According to a study, oxidative degradation of EVOO samples stored in transparent bottles exposed to light and those stored in closed bottles in the dark were investigated. The EVOO samples stored in transparent bottles exhibited higher FFA values than those stored in the dark, and the results indicated that exposure to sunlight caused oxidative degradation of EVOO (Kishimoto 2019).

**TABLE 3:** FFA, peroxide, and p‐AV value changes in oleogel and extra virgin olive oil during storage.

Property	Sample	Storage time			
		0	30	60	90
FFA (% linoleik)	LO	0.87 ± 0.07^Ac^	9.24 ± 0.12^Ab^	10.89 ± 0.57^Aa^	10.95 ± 0.06^Aa^
	DO	0.87 ± 0.07^Ac^	9.10 ± 0.06^Ab^	9.66 ± 0.74^ABb^	10.78 ± 0.37^Aa^
	LOO	0.78 ± 0.08^Ad^	7.57 ± 0.02^Bc^	8.93 ± 0.17^BCb^	9.69 ± 0.66^Ba^
	DOO	0.78 ± 0.08^Ab^	7.60 ± 0.22^Ba^	8.13 ± 1.02^Ca^	8.52 ± 0.65^Ca^
PV (meq O_2_ kg^−1^)	LO	0.62 ± 0.31^Ac^	23.01 ± 1.46^Bb^	26.16 ± 1.91^Bab^	30.45 ± 3.91^Ba^
	DO	0.62 ± 0.31^Ac^	14.19 ± 0.76^Cb^	16.48 ± 0.38^Ca^	16.63 ± 0.93^Ca^
	LOO	0.48 ± 0.21^Ad^	30.63 ± 2.16^Ac^	46.28 ± 1.51^Ab^	71.57 ± 0.65^Aa^
	DOO	0.48 ± 0.21^Ac^	13.15 ± 1.73^Cb^	13.57 ± 0.41^Dab^	16.31 ± 2.48^Ca^
p‐AV	LO	1.64 ± 0.09^Ad^	4.85±0.88^Bc^	5.94±0.35^Bb^	12.51±0.40^Ba^
	DO	1.64 ± 0.09^Ac^	2.65 ± 0.68^Cc^	4.83 ± 0.82^Cb^	11.19 ± 1.63^Ca^
	LOO	0.55 ± 0.38^Bd^	6.37 ± 0.44^Ac^	7.68 ± 0.52^Ab^	13.75 ± 0.33^Aa^
	DOO	0.55 ± 0.38^Bd^	5.40 ± 0.97^ABc^	6.54 ± 0.39^Bb^	13.23 ± 0.42^ABa^

*Note*: For each column, mean values of same capital letters are not statistically significant among applications (*p* > 0.05). For each line, mean values of same small letters are not statistically significant during storage (*p* > 0.05). LO: Light‐stored oleogel, DO: Dark‐stored oleogel, LOO: Light‐stored extra virgin olive oil, DOO: Dark‐stored extra virgin olive oil.

Upon analysis of the PVs of the samples, it was observed that values increased in all samples during storage (p < 0.05) (Table 3). This finding suggests the occurrence of primary oxidation in the samples during the storage period. The highest PV value was found in the LOO sample on all analysis days, indicating that light exposure accelerates oxidation. Conversely, the lowest PV values were recorded in the DO and DOO samples after 90 days, suggesting that storage in darkness and the gel structure demonstrated higher stability against primary oxidation. These values remained below 20 meq kg⁻¹, the threshold for significant sensory deterioration and poor quality (Thakur et al. 2023). This result supports the hypothesis that the gel structure and storage conditions can influence oxidative stability. The higher PVs observed in light‐exposed samples further support the protective effect of both darkness and the oleogel network. These findings are in line with the oxidative stability trend seen during frying, reinforcing the structural advantage of oleogels in slowing degradation pathways.

A study investigating olive oil oleogels reported that the type of wax used and storage temperature significantly influenced oxidative stability. It was observed that the PVs of oleogels remained lower than those of pure olive oil throughout the storage period, suggesting that gelation can reduce oxidative degradation and potentially extend shelf life (Dimakopoulou‐Papazoglou et al. 2024). Another study examined the oxidative stability of oleogels obtained using different gelators and macadamia oil during storage. The results showed that the PVs of the gelled samples were lower than those of the oil, indicating that gelation effectively reduced lipid oxidation and improved oxidative stability (Shuai et al. 2024). A similar study reported that oleogels increased peroxide stability compared to conventional palm oil during 90 days of storage (Noonim et al. 2022). The results of the present study are consistent with the findings reported in the extant literature, providing further evidence for the benefits of oleogels in enhancing oxidative stability.

The p‐AV value, an indicator of secondary oxidation, was measured on the analysis days during the storage period, and the results are presented in Table 3. At the initial stage (Day 0), p‐AV values were observed to be elevated in oleogel samples in comparison to oil samples (*p* < 0.05). During oleogel production, the oil is subjected to thermal or mechanical treatments, which may result in exposure to oxygen. Due to this interaction, the observed difference in p‐AV values between the EVOO and oleogel samples may have occurred as early as day 0. On all other days of analysis, the lowest p‐AV value was measured in oleogel samples stored under conditions of darkness DO.

In contrast, the highest p‐AV value was observed in oil samples stored under conditions of light (LOO) (*p* < 0.05). When comparing oleogel and oil samples stored under the same conditions, the p‐AV values of the oleogel samples were lower on all days of analysis. In addition, when comparing oleogel samples stored in dark and light conditions, the p‐AV values of the dark‐stored samples were lower throughout the analysis period. Considering the findings, it can be concluded that the gel structure and storage under dark conditions are more effective in slowing down secondary oxidation. In studies examining the effect of secondary oxidation in both oil and oleogel samples, it has similarly been observed that the gel structure delays the formation of secondary oxidation products (Hamidioglu et al. 2022; Hyatt et al. 2023; Kwon and Chang 2022).

In a study comparing the oxidative stability of saffron‐enriched sunflower oil stored under different conditions, it was observed that samples stored under conditions of darkness gave lower p‐AV values and behaved more stable against oxidation than those stored under conditions of light (Ahmed et al. 2024). In a separate study, the storage of sunflower oil enriched with rosemary and marjoram was conducted under conditions of darkness and light. The p‐AV value of the oil samples stored in light conditions was higher than that stored in darkness (Sahunie 2024). These results, which demonstrate the negative impact of light on oxidative stability, are consistent with our own findings. Moreover, the structural barrier provided by the oleogel matrix appears to enhance oxidative stability, particularly under dark storage conditions. To the best of our knowledge, no studies have investigated the effect of light on the storage stability of oleogels.

### 3.4 In Vitro Digestion Model

#### 3.4.1 Lipolysis

**FIGURE 5:**
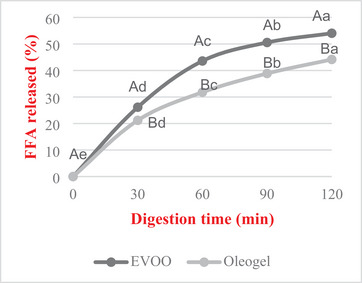
Total FFA release during digestion. Mean values of the same capital letters are not statistically significant among applications (p > 0.05). Mean values of the same small letters are not statistically significant during digestion (p > 0.05). EVOO: Extra Virgin Olive Oil.

The percentage of FFA increased significantly with time during small intestinal digestion (*p* < 0.05). This finding indicates that triacylglycerols are digested by lipase. It was observed that both EVOO and oil underwent rapid hydrolysis within 60 min, after which lipolysis gradually slowed down (Figure 5). A statistically significant lower FFA release rate was observed in oleogel compared to EVOO samples after the small intestinal digestion process (*p* < 0.05). The lipolysis percentages of EVOO and oleogel were 54% and 44%, respectively. The observed reduction in lipolysis (*p* < 0.05) in oleogel samples may be attributed to the dense gel network, which limits the accessibility of lipase to the oil phase. This interpretation is supported by Xu et al. (2024), who reported similar findings in structurally rigid oleogels. The formation of a strong gel network, as evidenced by the G′ > G″ values and low flow behavior index (*n*), is not only indicative of structural stability but may also contribute to this effect by acting as a physical barrier to digestive enzymes.

In a study, researchers compared the degree of lipolysis between diosgenin‐based oleogels and oils containing various unsaturated fatty acids. While the lipolysis of oils varied between 56% and 62%, the lipolysis of oleogels was between 33% and 44%. This result indicates that oleogels exhibit a significantly lower degree of lipolysis than oils (Lu et al. 2023). Another study compared the lipid digestion of sunflower oil and oleogels obtained using different oleogelators (monoglyceride, rice wax, phytosterol). Lipid digestion in the oil was higher than in the oleogels (54%). The lipolysis rate of the oleogels ranged from 32.8% to 45.1%. As a result, it was reported that different oil gelators and gelation mechanisms affect lipid digestion at varying levels (Calligaris et al. 2020). A study of the digestion of oleogel obtained with EVOO using lipophilic and hydrophilic gelators found that lipophilic gelators decreased lipolysis (Ciuffarin et al. 2023). Thus, it is possible to modulate lipid digestion by using specific gelators. Lipophilic oleogel can limit lipid digestion, thereby reducing calorie intake for consumers. Moreover, the ability of oleogels to reduce lipolysis may offer a dietary advantage by modulating fat digestion and potentially lowering energy absorption, which is of particular interest in developing functional foods for weight management. In contrast, using a hydrophilic oleogel will aid in effectively delivering essential fatty acids (Ciuffarin et al. 2023). However, further research is needed to understand the digestibility of oleogels fully.

#### 3.4.2 Tocopherol Bioaccessibility

**FIGURE 6:**
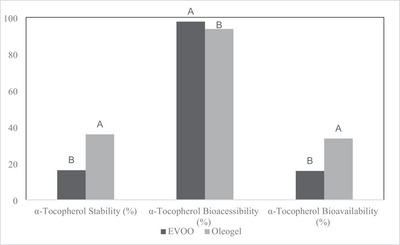
α‐tocopherol digestion stability, bioaccessibility, and estimated bioavailability. EVOO: Extra virgin olive oil. Mean values of the same capital letters are not statistically significant among applications (*p* > 0.05).

Bio accessibility is defined as the percentage of bioactive components that pass from the lipid phase to the aqueous phase after the mouth, stomach, and small intestine stages in the in vitro digestion process. The bioactive component is first released from the matrix and dissolved in the mixed micelle phase. This component, encapsulated only in the micelle phase, can be absorbed and utilized by intestinal epithelial cells during intestinal digestion (Shuai et al. 2024). Bioavailability refers to the amount of a compound available for use or storage in the body after digestion. This study evaluated bioavailability to estimate α‐tocopherol absorption, considering the interaction between stable and bio accessible bioactive compounds (Fernandes et al. 2024). Figure 6 presents the stability, bio accessibility, and bioavailability percentages of α‐tocopherol. Initially, the amount of α‐tocopherol in oleogel is lower than in EVOO. This observation can be attributed to oil exposure to oxygen and elevated temperatures during the manufacturing process of oleogel.

Following the conclusion of in vitro digestion, a substantial decline in the concentration of α‐tocopherol was evident in the oil and oleogel samples. Consequently, the stability and bioavailability of the samples were found to be low. The stability values were 16.21% for EVOO and 35.87% for the oleogel, while bioavailability was determined as 15.83% and 33.59%, respectively. Both parameters were significantly higher in the oleogel sample compared to EVOO (*p* < 0.05). This can be attributed to the negative impact of gastric acidity (pH = 3) on α‐tocopherol. The gel structure may have enhanced protection for α‐tocopherol compared to the effects of gastric acidity. During in vitro digestion, lipid oxidation may occur in addition to lipase activity (Alberdi‐Cedeño et al. 2020). Oleogels are known to exhibit more excellent resistance to oxidation compared to EVOO. This increased oxidative stability may explain the higher loss of α‐tocopherol observed in EVOO during digestion. Although oleogels exhibit more excellent oxidation resistance, the natural waxes used as gelling agents in oleogel production are believed to resist lipase activity. Therefore, they may possess poor digestibility characteristics (Okuro et al. 2021). In a study investigating the digestibility of rice bran wax oleogels, differences were observed in the fatty acid profiles of serum, liver, and feces of oleogel‐fed rats compared to the control group. It was reported that the gel structure reduced lipid digestion and absorption (Limpimwong et al. 2017). Therefore, alternative oleogelators instead of natural waxes as gelling agents may enhance bioavailability.

Bioavailability was high in both samples (EVOO 97.65%, oleogel 93.63%). Although the amount of α‐tocopherol decreased in both EVOO and oleogel after digestion, the remaining α‐tocopherols, which did not degrade in the digestate, transitioned to the micellar phase at a high rate, leading to the observed high bioavailability. Further studies are needed to enhance the bioavailability of α‐tocopherol after digestion. These studies may include shortening the time spent at elevated temperatures during oleogel preparation to reduce oxidation development and eliminating oxygen by applying a vacuum or filling the headspace with nitrogen during production. However, implementing such methods has the potential to increase the product's production costs (Alongi et al. 2022).

A further study could involve enriching oil and oleogel with phenolic compounds to reduce oxidation during the in vitro digestion phase. A study reported that the enrichment of olive oils with phenolic compounds reduced the degree of oxidation produced during in vitro digestion to minimum values or almost completely inhibited it (Alberdi‐Cedeño et al. 2020). Enriching oils and oleogels with α‐tocopherol represents a potential method for increasing bioavailability. The study aimed to replicate conventional fats' rheological properties and enhance the oil's nutritional value by incorporating α‐tocopherol into oleogel structured with CDW. The study concluded that the addition of α‐tocopherol did not adversely affect the rheological properties (Peixoto et al. 2024). A separate study investigated the textural and organoleptic properties of oleogels produced using α‐tocopherol, soybean oil, and monostearin. It was reported that these oleogels can be used as a substitute for hydrogenated and solid oils.

Furthermore, it was posited that these oleogels may be efficacious in increasing the bioavailability of α‐tocopherol (Monto et al. 2023). However, in vitro digestion studies of α‐tocopherol‐enriched oleogels are insufficient. While the addition of α‐tocopherol does not adversely affect structural and organoleptic properties, further investigation is required to ascertain its effect on bioavailability following digestion.

#### 3.4.3 Β‐Carotene Bioaccessibility

The β‐carotene released during digestion and transitioning to the micelle fraction represents the bioaccessible portion (Qiang et al. 2024). After in vitro digestion, β‐carotene bioaccessibility was 52.63% for oil and 40.91% for oleogel. The bioaccessibility rate is contingent upon the lipophilic‐hydrophilic balance character of the gelling agent employed (Ramezani et al. 2024). As the hydrophilic structure increases, digestive enzymes can penetrate the gel structure more effectively, increasing the bioaccessibility rate. The lower bioaccessibility of β‐carotene in oleogel compared to EVOO (*p* < 0.05) can be attributed to the lipophilic structure of CDW utilized in the present study. In a study, it was reported that the rate of lipolysis and the release of β‐carotene are proportional since the available micelle volume for the solubilization of β‐carotene increases with lipolysis (O′Sullivan et al. 2017). The findings of the present study were consistent with this information.

The wax employed in oleogel structuring exerts an influence on the rate of digestion by forming a crystalline network surrounding the oil phase, thereby reducing the potential bioavailability of the lipophilic compound and its absorption through the intestinal epithelium (Martins et al. 2025).

Bioavailability was found to be 6.2% and 2.8% for EVOO and oleogel, respectively, while stability was calculated as 11.53% and 7.17%, respectively. The amount of β‐carotene decreased significantly after digestion in both samples (*p* < 0.05). The total amount of bioactive compounds in the small intestine may be lower than the ingested amount due to chemical or biochemical transformations occurring in the mouth, stomach, or small intestine (McClements 2018). β‐carotene may undergo a series of degradation reactions in the stomach's acidic environment (Qiang et al. 2024). This decrease in β‐carotene can be associated with certain transformations and degradation reactions occurring during digestion.

In a study investigating the stability and bioaccessibility of β‐carotene, a zein‐oleogel structure was formed by dissolving β‐carotene in oleogel obtained from palm oil and monoglycerin. The study established that the bioaccessibility of β‐carotene in oil was 27%, 43%, and 46% in oil, oleogel, and zein‐oleogel, respectively (Qiang et al. 2024). In a separate study, the bioaccessibility of β‐carotene from sunflower oil, sterol‐based oleogels, and wax‐based oleogels was determined to be 3.3%, 4.0%, and 2.6%, respectively (Martins et al. 2025). As can be inferred from this, the bioaccessibility of β‐carotene can vary depending on numerous factors, such as the type of gel matrix used and differences in processing technologies.

## 4 Conclusions

The present study investigated the suitability of oleogel obtained with EVOO and CDW as a frying medium and its 90‐day storage stability. The results demonstrated that oleogel offered a more stable frying medium than EVOO while exhibiting reduced oil absorption in fried potatoes and enabling the production of lower‐calorie products. During the storage period, it was observed that stability against oxidation was enhanced, particularly in the dark and in the gel structure. During in vitro digestion, the gel structure enveloped the fat molecules, thereby reducing lipase and fat interaction. Consequently, the rate of lipolysis decreased, and the bioaccessibility of α‐tocopherol and β‐carotene decreased accordingly. Hydrophilic oleogelators in oleogel may increase the lipolysis rate and could effectively enhance the bio accessibility of bioactive compounds. In conclusion, the multifunctional advantages of olive oil‐based CDW oleogels, including improved frying performance, oxidative stability, and modulation of lipid digestion indicate strong potential for their application in the food industry, particularly in the formulation of healthier and more stable fat‐based products.

## Author Contributions

**Elif Kutahneci**: writing – original draft, visualization, validation, investigation, formal analysis, conceptualization. **Hasan Yalcin**: writing – review and editing, supervision, resources, project administration, conceptualization.

## Conflicts of Interest

The authors declare no conflicts of interest.

## Data Availability

Data will be made available upon reasonable request.

## References

[jfds70405-bib-0002] Adrah, K. , S. C. Adegoke , K. Nowlin , and R. Tahergorabi . 2022. “Study of Oleogel as a Frying Medium for Deep‐Fried Chicken.” Journal of Food Measurement and Characterization 16, no. 2: 1114–1123. 10.1007/s11694-021-01237-6.

[jfds70405-bib-0003] Ahmed, M. N. , K. Abourat , J. Gagour , E. H. Sakar , K. Majourhat , and S. Gharby . 2024. “Saffron (*Crocus sativus* L.) Stigmas as a Potential Natural Additive to Improve Oxidative Stability Attributes of Sunflower (*Helianthus annuus* L.) Oil Stored Under Different Conditions.” Grain & Oil Science and Technology 7, no. 3: 133–149. 10.1016/j.gaost.2024.06.001.

[jfds70405-bib-0004] Alberdi‐Cedeño, J. , M. L. Ibargoitia , and M. D. Guillén . 2020. “Study of the in Vitro Digestion of Olive Oil Enriched or Not With Antioxidant Phenolic Compounds. Relationships Between Bioaccessibility of Main Components of Different Oils and Their Composition.” Antioxidants 9, no. 6: 6. 10.3390/antiox9060543. PMC7346224 32575754

[jfds70405-bib-0005] Alongi, M. , P. Lucci , M. L. Clodoveo , F. P. Schena , and S. Calligaris . 2022. “Oleogelation of Extra Virgin Olive Oil by Different Oleogelators Affects the Physical Properties and the Stability of Bioactive Compounds.” Food Chemistry 368: 130779, 10.1016/j.foodchem.2021.130779. 34411852

[jfds70405-bib-0001] Anonymous . 1987. Standard Methods for the Analysis of Oils, Fats, and Derivatives: 1st Supplement to 7th Edition, 1st supplement to 7th edition. Pergamon Press.

[jfds70405-bib-0006] AOAC . 1998. Official Methods and Recommended Practices of the AOCS. AOCS.

[jfds70405-bib-0007] AOAC . 2000. Official Methods of Analysis of AOAC International, 17. AOAC International.

[jfds70405-bib-0008] Ash, D. , S. Majee , and G. R. Biswas . 2021. “Effect of Organogelator Concentration on Gelation Kinetics and Drug Release Behaviour of Paracetamol‐Loaded Soybean Oleogels.” International Journal of Pharmaceutical Sciences Review and Research 71, no. 2: 76–82. 10.47583/ijpsrr.2021.v71i02.013.

[jfds70405-bib-0009] Ashkar, A. , S. Laufer , J. Rosen‐Kligvasser , U. Lesmes , and M. Davidovich‐Pinhas . 2019. “Impact of Different Oil Gelators and Oleogelation Mechanisms on Digestive Lipolysis of Canola Oil Oleogels.” Food Hydrocolloids 97: 105218, 10.1016/j.foodhyd.2019.105218.

[jfds70405-bib-0010] Aydeniz Guneser, B. , E. Yılmaz , and E. K. Uslu . 2021. “Sunflower Oil–Beeswax Oleogels Are Promising Frying Medium for Potato Strips.” European Journal of Lipid Science and Technology 123, no. 10: 2100063, 10.1002/ejlt.202100063.

[jfds70405-bib-0011] Barragán‐Martínez, L. P. , L. Alvarez‐Poblano , E. J. Vernon‐Carter , and J. Alvarez‐Ramirez . 2022. “Effects of β‐carotene on the Color, Textural, Rheological and Structural Properties of Canola Oil/Beeswax Oleogel.” Journal of Food Measurement and Characterization 16, no. 5: 3946–3956. 10.1007/s11694-022-01449-4.

[jfds70405-bib-0012] Çakır, M. , C. O. Özer , and G. B. Var . 2023. “Utilization of Sunflower Oil‐Based Oleogel fordeep‐Fried Coated Chicken Products.” Journal of Oleo Science 72, no. 4: 399–407. 10.5650/jos.ess22365. 36990748

[jfds70405-bib-0013] Calligaris, S. , M. Alongi , P. Lucci , and M. Anese . 2020. “Effect of Different Oleogelators on Lipolysis and Curcuminoid Bioaccessibility Upon *in Vitro* Digestion of Sunflower Oil Oleogels.” Food Chemistry 314: 126146, 10.1016/j.foodchem.2019.126146. 31954944

[jfds70405-bib-0014] Chaturvedi, D. , D. Bharti , S. Dhal , et al. 2023. “Role of Stearic Acid as the Crystal Habit Modifier in Candelilla Wax‐Groundnut Oil Oleogels.” ChemEngineering 7, no. 5: 96. 10.3390/chemengineering7050096.

[jfds70405-bib-0015] Chauhan, D. S. , A. Khare , A. B. Lal , and R. P. Bebartta . 2022. “Utilising Oleogel as a Frying Medium for Deep Fried Indian Traditional Product (Mathri) to Reduce Oil Uptake.” Journal of the Indian Chemical Society 99, no. 3: 100378, 10.1016/j.jics.2022.100378.

[jfds70405-bib-0016] Chen, X. , S. Ding , Y. Chen , D. Lan , W. Wang , and Y. Wang . 2023. “Assessing the Effectiveness of Peanut Diacylglycerol Oil‐ethylcellulose/Monoglyceride‐Based Oleogel in Sponge Cake as a Margarine Replacer.” Food Bioscience 55: 102959, 10.1016/j.fbio.2023.102959.

[jfds70405-bib-0017] Ciuffarin, F. , M. Alongi , D. Peressini , L. Barba , P. Lucci , and S. Calligaris . 2023. “Role of the Polyphenol Content on the Structuring Behavior of Liposoluble Gelators in Extra Virgin Olive Oil.” Food Chemistry 412: 135572, 10.1016/j.foodchem.2023.135572. 36724719

[jfds70405-bib-0018] Ciuffarin, F. , M. Alongi , S. Plazzotta , et al. 2023. “Oleogelation of Extra Virgin Olive Oil by Different Gelators Affects Lipid Digestion and Polyphenol Bioaccessibility.” Food Research International 173: 113239, 10.1016/j.foodres.2023.113239. 37803552

[jfds70405-bib-0019] Dassanayake, L. S. K. , D. R. Kodali , S. Ueno , and K. Sato . 2009. “Physical Properties of Rice Bran Wax in Bulk and Organogels.” Journal of the American Oil Chemists' Society 86, no. 12: 1163–1173. 10.1007/s11746-009-1464-6.

[jfds70405-bib-0020] Demirkesen, I. , and B. Mert . 2020. “Recent Developments of oleogel Utilizations in Bakery Products.” Critical Reviews in Food Science and Nutrition 60, no. 14: 2460–2479. 10.1080/10408398.2019.1649243. 31385718

[jfds70405-bib-0021] Dimakopoulou‐Papazoglou, D. , K. Zampouni , P. Prodromidis , T. Moschakis , and E. Katsanidis . 2024. “Microstructure, Physical Properties, and Oxidative Stability of Olive Oil Oleogels Composed of Sunflower Wax and Monoglycerides.” Gels 10, no. 3: 195. 10.3390/gels10030195. 38534613 PMC10969988

[jfds70405-bib-0022] Doan, C. D. , I. Tavernier , P. K. Okuro , and K. Dewettinck . 2018. “Internal and External Factors Affecting the Crystallization, Gelation and Applicability of Wax‐Based Oleogels in Food Industry.” Innovative Food Science & Emerging Technologies 45: 42–52. 10.1016/j.ifset.2017.09.023.

[jfds70405-bib-0023] Dursun Capar, T. , and H. Yalcin . 2017. “Effects of Pre‐Drying on the Quality of Frying Oil and Potato Slices.” Quality Assurance and Safety of Crops and Foods 9, no. 3: 255–264. 10.3920/QAS2015.0691.

[jfds70405-bib-0024] Espert, M. , Q. Wang , T. Sanz , and A. Salvador . 2023. “Sunflower Oil‐Based Oleogel as Fat Replacer in Croissants: Textural and Sensory Characterisation.” Food and Bioprocess Technology 16, no. 9: 1943–1952. 10.1007/s11947-023-03029-w.

[jfds70405-bib-0025] Farooq, S. , M. I. Ahmad , Y. Zhang , M. Chen , and H. Zhang . 2023. “Preparation, Characterization and Digestive Mechanism of Plant‐Derived Oil Bodies‐Based Oleogels Structured by Chitosan and Vanillin.” Food Hydrocolloids 136: 108247, 10.1016/j.foodhyd.2022.108247.

[jfds70405-bib-0026] Fernandes, J. M. , J. F. Araújo , R. F. S. Gonçalves , A. A. Vicente , and A. C. Pinheiro . 2024. “Emulsions vs Excipient Emulsions as α‐tocopherol Delivery Systems: Formulation Optimization and Behaviour Under *in Vitro* Digestion.” Food Research International 192: 114743, 10.1016/j.foodres.2024.114743. 39147549

[jfds70405-bib-0027] Ghazani, S. M. , S. Dobson , and A. G. Marangoni . 2022. “Hardness, Plasticity, and Oil Binding Capacity of Binary Mixtures of Natural Waxes in Olive Oil.” Current Research in Food Science 5: 998–1008. 10.1016/j.crfs.2022.06.002. 35755304 PMC9213233

[jfds70405-bib-0029] Giuffrè, A. M. , C. Zappia , and M. Capocasale . 2017. “Effects of High Temperatures and Duration of Heating on Olive Oil Properties for Food Use and Biodiesel Production.” Journal of the American Oil Chemists' Society 94, no. 6: 819–830. 10.1007/s11746-017-2988-9.

[jfds70405-bib-0028] Giuffrè, A. M. , M. Caracciolo , C. Zappia , M. Capocasale , and M. Poiana . 2018. “Effect of Heating on Chemical Parameters of Extra Virgin Olive Oil, Pomace Olive Oil, Soybean Oil and Palm Oil.” Italian Journal of Food Science 30, no. 4:, 10.14674/IJFS-1269.

[jfds70405-bib-0030] Hamidioglu, I. , G. Alenčikienė , M. Dzedulionytė , A. Zabulionė , A. Bali , and A. Šalaševičienė . 2022. “Characterization of the Quality and Oxidative Stability of Hemp‐Oil‐Based Oleogels as an Animal Fat Substitute for Meat Patties,” Foods 11, no. 24: 4030. 10.3390/foods11244030. 36553772 PMC9778192

[jfds70405-bib-0031] Hosseini, H. , M. Ghorbani , N. Meshginfar , and A. S. Mahoonak . 2016. “A Review on Frying: Procedure, Fat, Deterioration Progress and Health Hazards.” Journal of the American Oil Chemists' Society 93, no. 4: 445–466. 10.1007/s11746-016-2791-z.

[jfds70405-bib-0032] Hyatt, J. R. , S. Zhang , and C. C. Akoh . 2023. “Characterization and Comparison of Oleogels and Emulgels Prepared From Schizochytrium Algal Oil Using Monolaurin and MAG/DAG as Gelators.” Journal of the American Oil Chemists' Society 100, no. 12: 945–959. 10.1002/aocs.12721.

[jfds70405-bib-0033] Igenbayev, A. , G. Ospankulova , S. Amirkhanov , A. Aldiyeva , I. Temirova , and K. Amirkhanov . 2023. “Substitution of Pork Fat With Beeswax‐Structured Oleogels in Semi‐Smoked Sausages.” Applied Sciences 13, no. 9: 9. 10.3390/app13095312.

[jfds70405-bib-0034] Jing, X. , Z. Chen , Z. Tang , et al. 2022. “Preparation of Camellia Oil Oleogel and Its Application in an Ice Cream System.” LWT 169: 113985, 10.1016/j.lwt.2022.113985.

[jfds70405-bib-0035] Keskin Uslu, E. , and E. Yılmaz . 2021. “Preparation and Characterization of Oleogels and Emulgels With Glycerol Monooleate–Cholesterol Mixtures.” Chemical Papers 75, no. 5: 2075–2085. 10.1007/s11696-020-01468-9.

[jfds70405-bib-0036] Khattab, R. 2022. “Physicochemical Quality and Thermal Stability of Vegetable Oils During Deep‐Fat Frying of Potato Chips.” Current Nutrition and Food Science 18: 337–348. 10.2174/1573401318666220903105129.

[jfds70405-bib-0037] Kishimoto, N. 2019. “Influence of Exposure to Sunlight on the Oxidative Deterioration of Extra Virgin Olive Oil During Storage in Glass Bottles.” Food Science and Technology Research 25, no. 4: 539–544. 10.3136/fstr.25.539.

[jfds70405-bib-0038] Kwon, U.‐H. , and Y. H. Chang . 2022. “Rheological and Physicochemical Properties of Oleogel With Esterified Rice Flour and its Suitability as a Fat Replacer.” Foods 11, no. 2: 242. 10.3390/foods11020242. 35053975 PMC8774694

[jfds70405-bib-0039] Li, X. , B. Zhao , Y. Zou , et al. 2024. “Structure, Rheology and Stability of Walnut Oleogels Structured by Cellulose Nanofiber of Different Lengths.” Food Hydrocolloids 154: 110148, 10.1016/j.foodhyd.2024.110148. 38977050

[jfds70405-bib-0040] Lim, J. , S. Jeong , I. K. Oh , and S. Lee . 2017. “Evaluation of Soybean Oil‐Carnauba Wax Oleogels as an Alternative to High Saturated Fat Frying Media for Instant Fried Noodles.” LWT 84: 788–794. 10.1016/j.lwt.2017.06.054.

[jfds70405-bib-0041] Limpimwong, W. , T. Kumrungsee , N. Kato , N. Yanaka , and M. Thongngam . 2017. “Rice Bran Wax Oleogel: A Potential Margarine Replacement and its Digestibility Effect in Rats Fed a High‐Fat Diet.” Journal of Functional Foods 39: 250–256. 10.1016/j.jff.2017.10.035.

[jfds70405-bib-0042] Liu, C. , Z. Zheng , Y. Shi , Y. Zhang , and Y. Liu . 2021. “Development of Low‐Oil Emulsion Gel by Solidifying Oil Droplets: Roles of Internal Beeswax Concentration.” Food Chemistry 345: 128811, 10.1016/j.foodchem.2020.128811. 33321346

[jfds70405-bib-0043] Lu, Y. , J. Li , J. Ding , X. Nie , N. Yu , and X. Meng . 2023. “Comparison of Diosgenin‐Vegetable Oils Oleogels With Various Unsaturated Fatty Acids: Physicochemical Properties, in‐Vitro Digestion, and Potential Mechanism.” Food Chemistry 413: 135663, 10.1016/j.foodchem.2023.135663. 36796264

[jfds70405-bib-0044] Lv, S. , Y. Zhang , H. Tan , R. Zhang , and D. J. McClements . 2019. “Vitamin E Encapsulation Within Oil‐in‐Water Emulsions: Impact of Emulsifier Type on Physicochemical Stability and Bioaccessibility.” Journal of Agricultural and Food Chemistry 67, no. 5: 1521–1529. 10.1021/acs.jafc.8b06347. 30663308

[jfds70405-bib-0045] Mahmud, N. , J. Islam , W. Oyom , K. Adrah , S. C. Adegoke , and R. Tahergorabi . 2023. “A Review of Different Frying Oils and Oleogels as Alternative Frying Media for Fat‐uptake Reduction in Deep‐Fat Fried Foods.” Heliyon 9, no. 11: e21500, 10.1016/j.heliyon.2023.e21500. 38027829 PMC10660127

[jfds70405-bib-0046] Manzoor, S. , F. A. Masoodi , F. Naqash , and R. Rashid . 2022. “Oleogels: Promising Alternatives to Solid Fats for Food Applications.” Food Hydrocolloids for Health 2: 100058, 10.1016/j.fhfh.2022.100058.

[jfds70405-bib-0047] Martins, A. J. , L. Perdigão , C. Gonçalves , et al. 2025. “Beta‐Carotene‐loaded Oleogels: Morphological Analysis, Cytotoxicity Assessment, *in Vitro* Digestion and Intestinal Permeability.” Food Chemistry 465: 142085, 10.1016/j.foodchem.2024.142085. 39571441

[jfds70405-bib-0048] McClements, D. J. 2018. “Enhanced Delivery of Lipophilic Bioactives Using Emulsions: A Review of Major Factors Affecting Vitamin, Nutraceutical, and Lipid Bioaccessibility.” Food and Function 9, no. 1: 22–41. 10.1039/C7FO01515A. 29119979

[jfds70405-bib-0049] Minekus, M. , M. Alminger , P. Alvito , et al. 2014. “A Standardised Static in Vitro Digestion Method Suitable for Food—an International Consensus.” Food and Function 5, no. 6: 1113–1124. 10.1039/C3FO60702J. 24803111

[jfds70405-bib-0050] Monto, A. R. , L. Yuan , Z. Xiong , et al. 2023. “α‐Tocopherol Stabilization by Soybean Oil and Glyceryl Monostearate Made Oleogel: Dynamic Changes and Characterization for Food Application.” LWT 187: 115325, 10.1016/j.lwt.2023.115325.

[jfds70405-bib-0051] Moon, K. , K.‐O. Choi , S. Jeong , Y.‐W. Kim , and S. Lee . 2021. “Solid Fat Replacement With Canola Oil‐Carnauba Wax Oleogels for Dairy‐Free Imitation Cheese Low in Saturated Fat.” Foods 10, no. 6: 1351. 10.3390/foods10061351. 34208054 PMC8230639

[jfds70405-bib-0079] Noonim, P. , B. Rajasekaran , and K. Venkatachalam . 2022. “Structural Characterization and Peroxidation Stability of Palm Oil‐Based Oleogel Made with Different Concentrations of Carnauba Wax and Processed with Ultrasonication.” Gels 8, no. 12: 763. 10.3390/gels8120763. 36547287 PMC9778256

[jfds70405-bib-0052] O′Sullivan, C. M. , M. Davidovich‐Pinhas , A. J. Wright , S. Barbut , and A. G. Marangoni . 2017. “Ethylcellulose Oleogels for Lipophilic Bioactive Delivery—Effect of Oleogelation on in Vitro Bioaccessibility and Stability of Beta‐Carotene.” Food and Function 8, no. 4: 1438–1451. 10.1039/C6FO01805J. 28345698

[jfds70405-bib-0054] Öğütcü, M. , and E. Yılmaz . 2015. “Comparison of the Pomegranate Seed Oil Organogels of Carnauba Wax and Monoglyceride.” Journal of Applied Polymer Science 132, no. 4:, 10.1002/app.41343.

[jfds70405-bib-0053] Öğütcü, M. , N. Arifoğlu , and E. Yılmaz . 2015. “Preparation and Characterization of Virgin Olive Oil‐Beeswax Oleogel Emulsion Products.” Journal of the American Oil Chemists' Society 92, no. 4: 459–471. 10.1007/s11746-015-2615-6.

[jfds70405-bib-0055] Okuro, P. K. , T. P. Santos , and R. L. Cunha . 2021. “Compositional and Structural Aspects of Hydro‐ and Oleogels: Similarities and Specificities From the Perspective of Digestibility.” Trends in Food Science and Technology 111: 55–67. 10.1016/j.tifs.2021.02.053.

[jfds70405-bib-0056] Pathare, P. B. , U. L. Opara , and F. A. Al‐Said ‐J. 2013. “Colour Measurement and Analysis in Fresh and Processed Foods: a Review.” Food and Bioprocess Technology 6, no. 1: 36–60. 10.1007/s11947-012-0867-9.

[jfds70405-bib-0057] Pehlivanoğlu, H. , M. Demirci , O. S. Toker , N. Konar , S. Karasu , and O. Sagdic . 2018. “Oleogels, a Promising Structured Oil for Decreasing Saturated Fatty Acid Concentrations: Production and Food‐based Applications.” Critical Reviews in Food Science and Nutrition 58, no. 8: 1330–1341. 10.1080/10408398.2016.1256866. 27830932

[jfds70405-bib-0058] Peixoto, V. O. D. S. , G. B. Brito , E. C. A. N. Chrisman , et al. 2024. “Tailoring Candelilla Wax‐based Oleogels Loaded With α‐tocopherol to Mimic Rheological Properties of Solid Food Fats and to Increase Nutritional Value of Food.” Journal of the American Oil Chemists' Society 102, no. 2: 279–293. 10.1002/aocs.12884.

[jfds70405-bib-0059] Pușcaș, A. , A. Mureșan , F. Ranga , et al. 2020. “Phenolics Dynamics and Infrared Fingerprints During the Storage of Pumpkin Seed Oil and Thereof Oleogel.” Processes 8, no. 11: 1412. 10.3390/pr8111412.

[jfds70405-bib-0060] Qiang, S. , J. Zhou , T. Yang , et al. 2024. “Structure, Stability and *in Vitro* Digestion of a Novel Zein‐Based Oil Gel Delivery System Loaded β‐Carotene.” Journal of Food Engineering 366: 111848, 10.1016/j.jfoodeng.2023.111848.

[jfds70405-bib-0061] Ramezani, M. , O. Martín‐Belloso , and L. Salvia‐Trujillo . 2024. “Influence of Oleogel Composition on Lipid Digestibility and β‐carotene Bioaccessibility During *in Vitro* Digestion.” Food Chemistry 456: 139978, 10.1016/j.foodchem.2024.139978. 38870810

[jfds70405-bib-0062] Romani, A. , F. Ieri , S. Urciuoli , et al. 2019. “Health Effects of Phenolic Compounds Found in Extra‐Virgin Olive Oil, by‐Products, and Leaf of Olea Europaea L.” Nutrients 11, no. 8: 1776. 10.3390/nu11081776. 31374907 PMC6724211

[jfds70405-bib-0078] Sabet, S. , S. J. Kirjoranta , A. M. Lampi , M. Lehtonen , E. Pulkkinen , and F. Valoppi . 2022. “Addressing Criticalities in the INFOGEST Static In Vitro Digestion Protocol for Oleogel Analysis.” Food Research International 160: 111633. 10.1016/j.foodres.2022.111633 36076373

[jfds70405-bib-0063] Sahunie, A. 2024. “Effect of *Rosmarinus officinalis* and *Origanum majorana* Extracts on Stability of Sunflower Oil During Storage and Repeated Heating.” Oil Crop Science 9, no. 1: 29–37. 10.1016/j.ocsci.2023.12.006.

[jfds70405-bib-0064] Shuai, X. , D. Julian McClements , T. Dai , et al. 2024. “Effect of Different Oleogelators on Physicochemical Properties, Oxidative Stability and Astaxanthin Delivery of Macadamia Oil‐Based Oleogels.” Food Research International 196: 115131, 10.1016/j.foodres.2024.115131. 39614525

[jfds70405-bib-0065] Singh, A. , F.‐I. Auzanneau , and M. A. Rogers . 2017. “Advances in Edible Oleogel Technologies—A Decade in Review.” Food Research International 97: 307–317. 10.1016/j.foodres.2017.04.022. 28578056

[jfds70405-bib-0067] Sivakanthan, S. , S. Fawzia , S. Mundree , T. Madhujith , and A. Karim . 2024. “Investigation of the Influence of Minor Components and Fatty Acid Profile of Oil on Properties of Beeswax and Stearic Acid‐Based Oleogels.” Food Research International 184: 114213, 10.1016/j.foodres.2024.114213. 38609212

[jfds70405-bib-0066] Sivakanthan, S. , S. Fawzia , T. Madhujith , and A. Karim . 2022. “Synergistic Effects of Oleogelators in Tailoring the Properties of Oleogels: A Review.” Comprehensive Reviews in Food Science and Food Safety 21, no. 4: 3507–3539. 10.1111/1541-4337.12966. 35591753

[jfds70405-bib-0068] Tajer, A. , and S. Ozdemir . 2023. “The Frying Stability of Oleogel Made From Rice Bran Wax and Sunflower Oil during Intermittent Frying of Potato Chips.” Journal of Oleo Science 72, no. 11: 997–1004. 10.5650/jos.ess22408. 37914269

[jfds70405-bib-0069] Thakur, D. , A. Singh , P. K. Prabhakar , M. Meghwal , and A. Upadhyay . 2022. “Optimization and Characterization of Soybean Oil‐Carnauba Wax Oleogel.” LWT 157: 113108, 10.1016/j.lwt.2022.113108.

[jfds70405-bib-0070] Thakur, D. , R. Suhag , A. Singh , A. Upadhyay , P. K. Prabhakar , and A. Sharma . 2023. “Comparative Evaluation of Soybean Oil‐Carnauba Wax Oleogel as an Alternative to Conventional Oil for Potato Chips Frying.” Food Structure 37: 100334, 10.1016/j.foostr.2023.100334.

[jfds70405-bib-0071] Xu, B. , X. Lin , Y. Zhao , et al. 2024. “The Effect of Citral Loading and Fatty Acid Distribution on the Oleogels: Physicochemical Properties and in Vitro Digestion.” Food Chemistry 459: 140337, 10.1016/j.foodchem.2024.140337. 38996640

[jfds70405-bib-0072] Yang, J. , H. Zheng , Y. Mo , Y. Gao , and L. Mao . 2022. “Structural Characterization of Hydrogel‐Oleogel Biphasic Systems as Affected by Oleogelators.” Food Research International 158: 111536, 10.1016/j.foodres.2022.111536. 35840233

[jfds70405-bib-0073] Yang, S. , X. Zhang , A. S. M. Saleh , L. Wang , Y. Duan , and Z. Xiao . 2024. “Effect of β‐sitosterol and Palmitic Acid Mass Ratio on Structural, Physicochemical, and Rheological Properties of Rice Bran Oil‐based Oleogel.” LWT 209: 116775, 10.1016/j.lwt.2024.116775.

[jfds70405-bib-0074] Yi, B. , M.‐J. Kim , S. Y. Lee , and J. Lee . 2017. “Physicochemical Properties and Oxidative Stability of Oleogels Made of Carnauba Wax With Canola Oil or Beeswax With Grapeseed Oil.” Food Science and Biotechnology 26, no. 1: 79–87. 10.1007/s10068-017-0011-8. 30263513 PMC6049465

[jfds70405-bib-0075] Yilmaz, E. , E. Keskin Uslu , and C. Öz . 2021. “Oleogels of some Plant Waxes: Characterization and Comparison With Sunflower Wax Oleogel.” Journal of the American Oil Chemists' Society 98, no. 6: 643–655. 10.1002/aocs.12490.

[jfds70405-bib-0076] Zhou, M. , B. Li , A. Wu , et al. 2025. “Preparation of a Two‐Phase Gel System Based on Gelatin Hydrogel and Beeswax/Rice Bran Wax Oleogel and Eutectic Phase Behavior of Beeswax and Rice Bran Wax in Soybean Oil.” LWT 215: 117306, 10.1016/j.lwt.2024.117306.

[jfds70405-bib-0077] Zubairee, K. , H. Yalcin , and T. Dursun Capar . 2024. “Sunflower Oil‐Soybean Wax Oleogel: an Oxidation Stable Alternative to Traditional Frying Methods for Doughnut.” Journal of the American Oil Chemists' Society 102, no. 2: 239–250. 10.1002/aocs.12882.

